# Ethnic Differences in Dementia Diagnosis and Treatment in Israel

**DOI:** 10.3390/jcm14175926

**Published:** 2025-08-22

**Authors:** Haya Bishara, Hilla Cohen, Dana Hadar, Polina Specktor

**Affiliations:** 1 Neurology Department, Carmel Medical Center, Haifa 3436212, Israel; poly.specktor@gmail.com; 2 Research Department, Carmel Medical Center, Haifa 3436212, Israel; hillaco@clalit.org.il (H.C.); cmddana@clalit.org.il (D.H.)

**Keywords:** dementia, Alzheimer’s disease, mild cognitive impairment

## Abstract

**Background/Objectives:** Lifestyle and socioeconomic disparities influence dementia prevalence and treatment across ethnic groups worldwide. Our study aimed to examine differences in the diagnosis, work-up, and treatment of mild cognitive impairment (MCI) as well as various types of dementia provided to Arab and Jewish participants in Israel. **Methods:** Data were collected retrospectively and anonymously between 1 January 2010 and 12 September 2021, by Clalit Health Services’ research department. Ethnicity was determined based on residence, including only cities with a 99% majority of either Jewish or Arab participants, according to the Central Bureau of Statistics (CBS). Subjects over the age of 60 with an MCI or dementia diagnosis were analyzed. **Results:** Of 212,091 diagnosed individuals, 14,742 were of a definite ethnicity as defined. The mean age at diagnosis was significantly younger for Arab participants (75.78 ± 10.28) compared to Jewish participants (80.14 ± 9.45) (*p* < 0.001). The gender distribution was similar (42.5% male). The most common diagnosis was Alzheimer’s disease (AD), affecting 2495 (29.8%) of Jewish participants and 2387 (38%) of Arab participants (*p* < 0.001). Vascular dementia (VD) was also more prevalent in Arab participants, 12.6%, vs. 6.42% in Jewish participants (*p* < 0.001). MCI was more common in Jewish participants, 26.2%, compared to 10.3% in Arab participants (*p* < 0.001). **Conclusions:** Arab participants were diagnosed with dementia at a younger age and showed higher rates of AD and VD diagnosis compared to Jewish participants, but were significantly less likely to be diagnosed with MCI. Efforts to understand and address these underlying causes are warranted to promote health equity.

## 1. Introduction

Dementia is a syndrome characterized by declines in cognitive, behavioral, social, and functional domains, and is one of the leading causes of disability and dependency among older people worldwide. With increasing longevity, the prevalence and burden of dementia are rising exponentially [1]. The early diagnosis of dementia is a critical healthcare priority, offering opportunities to identify causes, coordinate care, plan for the future, and enroll appropriate candidates in clinical trials [1].

An expanding body of research highlights that ethnic disparities in dementia are not isolated phenomena but are deeply embedded in broader social and structural inequities. The concept of Social Determinants of Health (SDOH)—the conditions in which people are born, live, learn, work, and age—is fundamental to understanding these patterns. Factors such as lower socioeconomic status (SES), lower levels of education, poverty, and exposure to discrimination are recognized as significant contributors to increased dementia risk [2]. Studies in various countries report that ethnic minorities often have higher rates of dementia. This increased risk is frequently mediated by lower socioeconomic status, higher rates of illiteracy, a greater prevalence of comorbidities (particularly cardiovascular risk factors), and limited access to health services, especially in their native language. Consequently, individuals from ethnic minorities are underrepresented in dementia care centers [1,3,4,5,6,7,8,9] and are typically diagnosed at a later stage of the disease, when symptoms are more severe and impairments are more pronounced [4,6,10,11,12].

Israel, a multi-ethnic society, provides a unique setting with which to study ethnic disparities in dementia. The population in Israel is predominantly Jewish (73%), with an Arab minority (21%) and 5% defined as “others” [13]. While few epidemiologic studies on dementia have been conducted in Israel, they have primarily estimated the prevalence of dementia separately within the Jewish and Arab populations. Kahana (2003) reported a dementia prevalence of 5.9% among individuals aged 76 and 26.9% among those over the age of 90 in the Jewish population [14]. Bowirrat (2001) found a dementia prevalence of 8% among individuals aged 60–69 and 51% among those over the age of 70 in the Arab population of Wadi Ara [15]. Another study, conducted in the same community in 2012, found that almost 10% had AD and 32.1% had mild cognitive impairment (MCI) [16]. The variability in these results can likely be attributed to differences in population selection and testing methodologies.

A 2020 study examining the representation of ethnic groups in a tertiary dementia clinic in Haifa, in the north of Israel, found that the Arab population constituted only 7.8% of those evaluated, despite comprising 37% of the northern Israeli population aged 55 and older and 17% of Haifa district residents [3]. Moreover, Arab subjects were referred at a younger age and at more advanced stages of cognitive decline, with lower MMSE scores and more severe functional impairment compared to their Jewish counterparts [3]. However, the scope of this research was limited, as it was performed in a single, private tertiary clinic and its findings may not be generalizable to Israel’s entire population. No ethnicity-based population comparative study has been performed in Israel to date.

This study aims to compare demographic and clinical features of dementia in Arab and Jewish subjects in Israel. To our knowledge, no previous study has comprehensively addressed this issue at the national level.

## 2. Methods

We used data from Clalit Health Services (CHS), Israel’s largest healthcare provider, which insured 5.2 million people in 2021, representing 53% of the country’s population. The database includes patient demographics and characteristics (e.g., age, gender, weight, and smoking status), medical diagnoses coded by the International Classification of Diseases (ICD), drug prescriptions and dispenses, referrals to consultants, laboratory and imaging results, and hospitalizations.

The study was approved by the Helsinki Committee of Carmel Hospital (approval number: 0171-21-COM). Data were retrieved via the Carmel Data Research Center (CDRS), which ensures anonymity through de-identification software. All data were securely stored on the MDClone cloud platform (https://www.mdclone.com), and statistical analyses were conducted on datasets accessed with permission from CHS security.

This was a retrospective cohort study. We searched the CHS database for individuals aged 60 and over with recorded diagnoses of dementia or MCI between 1 January 2010 and 12 September 2021. The specialty of the physician providing the dementia diagnosis was documented.

MCI was coded as an official MCI diagnosis or diagnosis of cognitive deficits, and memory loss without a dementia diagnosis. If etiology was mentioned, dementia was subtyped according to etiology: Alzheimer disease (AD), vascular dementia (VD), Parkinson‘s dementia (PD), and other types. If the dementia etiology was not mentioned, it was subtyped as dementia NOS (not otherwise specified).

Since ethnic data was missing from CHS, ethnicity was determined by the subjects’ place of residence, including only cities with a 99% majority of either Jewish or Arab residents, according to the Central Bureau of Statistics (CBS). Subsequently, socioeconomic state (SES) and education years (EYs) were also retrieved from the CBS based on place of residence.

Jewish and Arab participants over the age of 60 were assessed for dementia characteristics including demographic data, medical comorbidities, body mass index (BMI), and physical activity. Engagement in physical activity was defined based on patient self-reports. Any weekly performance of physical activity was classified as positive physical activity. Dementia work-ups (imaging and blood tests), treatments, and follow-ups were recorded.

Specifically, the following drugs that are approved for the treatment of dementia in Israel were assessed: Cerebonin (a dry extract from Ginkgo biloba leaves, approved for the treatment of MCI and dementia of the AD, VD, or mixed subtype), Rivastigmine (a central acetylcholinesterase inhibitor approved for the treatment of AD and PD), Galantamine and Donepezil (two other central acetylcholinesterase inhibitors approved for the treatment of AD), and Ebixa (an NMDA receptor antagonist used for moderate to severe AD). Anti-amyloid drugs were not evaluated as their cost is covered only by private insurance. Medication adherence was defined as filling prescriptions for at least three months of anti-dementia medication. Additionally, treatment with antipsychotic drugs, both typical and atypical (three or more issued prescriptions), was recorded as a means with which to evaluate the treatment of behavioral symptoms.

Socioeconomic data, including education years (EYs) and SES, were retrieved from the CBS based on the patient’s place of residence.

Statistical analysis: Descriptive statistics were used to characterize the study population. Categorical variables are presented as frequencies and percentages, while continuous variables are reported as means with standard deviations (SDs).

To compare demographic and clinical characteristics between the Jewish and Arab groups, univariate analyses were conducted. The chi-squared (χ^2^) test or Fisher’s exact test was used for categorical variables, and independent t-tests were used for continuous variables, with the choice of test depending on distributional assumptions.

To identify factors independently associated with ethnicity, a multivariable logistic regression model was constructed. The model used the population group (Arab vs. Jewish) as the dependent variable and included significant demographic and clinical characteristics as covariates. Odds ratios (ORs), with their corresponding 95% confidence intervals (CIs), were reported. A two-sided *p*-value < 0.05 was considered statistically significant. All analyses were performed using R (version 4.2.3).

## 3. Results

### 3.1. Demographics and Clinical Characteristics

A total of 212,091 individuals aged 60 and older were identified with a diagnosis of either MCI or one of the subtypes of dementia, covering the period from 1 January 2010 to 12 September 2021. Among these, 14,742 met the study’s criteria for ethnicity based on residence. The final cohort included 6275 Arab and 8467 Jewish participants.

The mean age at diagnosis differed significantly between groups: Arab participants had a mean age of 75.78 years (±10.28), while Jewish participants averaged 80.14 years (±9.45), (*p* < 0.001). The gender distribution was comparable, with males representing 42.5% of the cohort (Table 1).

### 3.2. Health Conditions and Risk Factors

Information regarding BMI, physical activity, and smoking status was available for only a subset of participants. The mean BMI was slightly higher in the Arab group, with a mean of 28.48 (±6.09), compared to 27.09 (±5.23) in the Jewish group (*p* < 0.001). Jewish participants reported higher levels of physical activity, with 1334 (20.9%) being active, compared to 550 (10.2%) of Arab participants (*p* < 0.001). Conversely, smoking was more prevalent in the Arab group, with 1821 (30%) being current or past smokers, compared to 2027 (25.8%) in the Jewish group (*p* < 0.001) (Table 1).

Cardiovascular risk factors of diabetes and hyperlipidemia were significantly more commonly diagnosed among Arab participants, with 2970 cases of diabetes (47.3%), versus 3009 (35.5%) for Jewish participants (*p* < 0.001), and 2390 (38.1%) cases of hyperlipidemia versus 2881 (34.0%), respectively (*p* < 0.001). A hypertension diagnosis was more prevalent among Jewish participants, with 4714 cases (55.7%) compared to 3218 (51.3%) in Arab participants (*p* < 0.001) (Table 1).

A history of malignancy was significantly more common in the Jewish group, affecting 2356 individuals (27.8%), compared to 687 (10.9%) in the Arab group (*p* < 0.001). Similarly, mood disorders, including depression and anxiety, were more commonly diagnosed in the Jewish population; depression was diagnosed in 2417 (28.5%) Jewish participants versus 985 (15.7%) Arab participants, while anxiety was diagnosed in 307 (3.6%) versus 114 (1.8%), respectively (both *p* < 0.001) (Table 1).

Intracerebral hemorrhage was diagnosed more frequently in the Arab group, occurring in 1879 individuals (29.9%), compared to 1977 (23.3%) in the Jewish group (*p* < 0.001). Diagnoses of ischemic stroke were similar between the ethnic groups, occurring in 2815 (33.2%) Jewish participants and 2142 (34.1%) Arab participants (*p* = 0.259) (Table 1).

### 3.3. Diagnosis and Initial Work-Up

**Figure 1:**
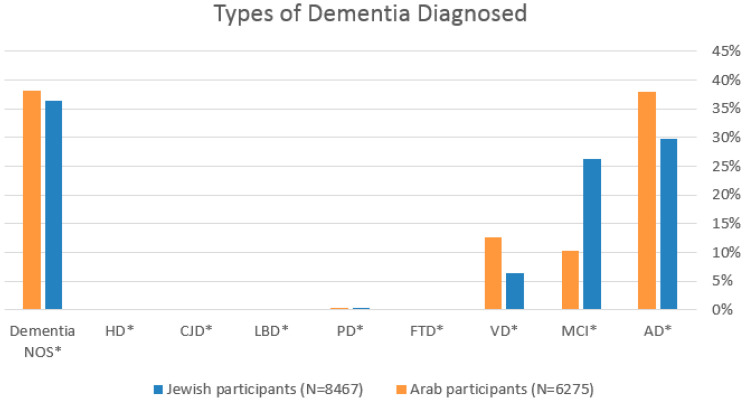
Distribution of dementia subtypes diagnosed among Arab and Jewish patients. Dementia NOS: not otherwise specified; * (HD: Huntington disease; CJD: Creutzfeldt–Jakob disease; LBD: Lewy Body Dementia; PD: Parkinson’s Disease; FTD: fronto-tempotal Dementia; VD: Vascular Dementia; MCI: Mild Cognitive Impairment; AD: Alzheimer’s Disease).

AD was the most frequent diagnosis; it was more common among Arab participants, affecting 2524 (29.8%) Jewish participants compared to 2387 (38%) Arab participants (*p* < 0.001). MCI was more prevalent in Jewish subjects (26.3%, *n* = 2227) versus Arab subjects (10.3%, *n* = 647) (*p* < 0.001). VD was more common in Arab participants (12.6%, *n* = 789) compared to Jewish participants (6.42%, *n* = 540) in (*p* < 0.001). Other forms of dementia, such as frontotemporal dementia, Lewy body dementia, and PD, were more prevalent among Jewish patients, although these differences were not statistically significant (Table 2, Figure 1).

Dementia or MCI was most often diagnosed by general practitioners (GPs), accounting for 68.2% (*n* = 3385) of diagnoses in Jewish participants and 76.1% (*n* = 2630) in Arab participants (*p* < 0.001). Jewish subjects were more frequently diagnosed by geriatricians (*n* = 876, 17.7%) compared to Arab subjects (*n* = 249, 7.2%) (*p* < 0.001). In contrast, Arab participants were more commonly diagnosed by neurologists (*n* = 397, 11.5%) compared to Jewish participants (*n* = 388, 7.8%) (*p* < 0.001) (Table 2).

An initial work-up involving head imaging (CT or MRI) was conducted at similar rates in both groups, for 50.6% (*n* = 4282) of Jewish participants and 49.3% (*n* = 3092) of Arab participants (*p* = 0.119). However, laboratory work-ups for vitamin B12 levels and thyroid-stimulating hormone (TSH) were more commonly performed in Jewish patients; vitamin B12 levels were checked in 84.5% of Jewish versus 62.8% of Arab participants, and TSH levels were checked in 95.0% of Jewish versus 85.3% of Arab participants (both *p* < 0.001). LDL measurements were obtained similarly across groups, whereas HbA1c testing was more frequently performed for Arab (*n* = 4737, 75.5%) than for Jewish participants (*n* = 5900, 69.7%) (*p* < 0.001) (Table 2).

### 3.4. Treatment and Follow-Up Care

Jewish participants were more likely to purchase medications for dementia (*n* = 2231, 82.4%) compared to Arab participants (*n* = 893, 73%) (*p* < 0.001). Jewish and Arab patients purchased antipsychotic medications at similar rates: 63.5% (*n* = 1634) versus 62.1% (*n* = 1350), respectively (*p* = 0.360) (Table 3).

Arab participants had more follow-up visits per year to both neurologists (averaging 1.97 ± 3.08 visits) and psychiatrists (2.707 ± 3.825 visits) compared to Jewish participants (1.502 ± 2.702 and 2.002 ± 3.179 visits, respectively; *p* < 0.001). However, Jewish participants had a greater average number of follow-up years, at 6.805 (±5.240) compared to 5.714 (±4.909) for Arab participants (*p* < 0.001) (Table 3). Residential characteristics: Data on residential characteristics from the CBS were available for 6393 participants. Socioeconomic status (SES) and educational attainment, according to the CBC, were notably lower among Arab participants. The mean SES score was 3.237 (±1.254) for Arab participants versus 6.436 (±3.126) for Jewish participants (*p* < 0.001). Mean educational years (EYs) were 12.004 (±0.633) for Arab participants compared to 13.917 (± 1.056) for Jewish participants (*p* < 0.001). Additionally, a higher proportion of Jewish participants held academic degrees (22.34% vs. 6.16%, *p* < 0.001) and reported a greater average monthly income (ILS 8082.21 ± 3788.06) compared to that of Arab participants (ILS 4094.81 ± 947.78) (*p* < 0.001) (Table 4).

#### Multivariable Analysis of Ethnicity and Dementia Subtype

To identify factors independently associated with ethnicity, a multivariable logistic regression model was constructed with ethnic group (Arab vs. Jewish) as the dependent variable. Covariates were selected for inclusion if they were clinically relevant or showed a significant association with the population group in univariate analyses (*p* < 0.1).

In the multivariable logistic regression, after adjusting for demographic data (Table 1), Arab ethnicity was significantly associated with an AD diagnosis (OR = 1.75, 95% CI [1.62–1.89]). This association remained significant even when further adjusting for residential and socioeconomic characteristics (Table 4) in the subsample with available CBS data (*n* = 6393), yielding an OR of 1.73 (95% CI [1.40–2.13]) (Tables S1 and S2 in the Supplemental Materials). For VD, Arab ethnicity was also associated with increased odds in the model adjusted only for demographics (OR = 1.35, 95% CI [1.18–1.53]). After adding residential and socioeconomic characteristics, this association was less statistically significant (OR = 1.39, 95% CI [0.96–2.03]) (Tables S3 and S4 in the Supplemental Materials). As a sensitivity analysis, the results from the primary model were compared to a complete-case analysis, which showed similar trends.

## 4. Discussion

Our study explored ethnic differences in the age of dementia diagnosis, its subtypes, and treatment among community-dwelling individuals in Israel.

Our findings indicate that Arab participants were diagnosed with dementia and MCI approximately four years earlier than Jewish participants. While AD and VD were more common among Arab patients, MCI was more prevalent among Jewish patients. Furthermore, Arab participants were less likely to undergo recommended dementia work-ups, such as vitamin B12 and TSH analyses. In contrast, Jewish participants had longer follow-up periods and more frequently purchased anti-dementia medications. In a multivariable logistic regression adjusted for demographic and socioeconomic factors, Arab ethnicity was significantly associated with an AD diagnosis.

These results suggest disparities in the characteristics, diagnosis, and treatment of dementia between Israel’s majority Jewish population and its Arab minority. Our findings are consistent with previous studies in Israel reporting a younger age at diagnosis and higher dementia prevalence, particularly AD, among the Arab population [3,13,16,17,18]. They also align with worldwide literature showing disparities in dementia diagnosis and treatment among ethnic minorities [1,3,4,5,6,7,8,9].

There may be several explanations and contributing factors to these findings.

First, according to the CBS, socioeconomic status and educational attainment were notably lower among Arab participants in the homogeneous Arab and Jewish cities that we included. Jewish participants had a greater monthly income, a higher mean of EYs, and a higher proportion of subjects with academic degrees [13]. Additionally, Arab patients had a higher prevalence of diabetes, hyperlipidemia, and higher BMI; they were also more likely to smoke and less likely to perform regular physical activity, all of which are risk factors for dementia [1,3,4,5,6,7,8].

Second, due to a lower level of education, Arab participants may have limited awareness regarding dementia, potentially perceiving it as a “normal part of aging” rather than a condition that needs treatment [3,4,5,7]. Moreover, they may have limited awareness of available services for dementia diagnosis and treatment, especially in their native language [1,3,4,5,6,7,8]. Finally, due to their lower education levels, they may be reluctant to diagnose and treat dementia due to cultural stigma surrounding it [3,4,5,7,19]. This explanation may be supported by our finding that Arab patients were more commonly diagnosed with AD and less commonly with MCI compared to Jewish patients.

Third, the lower socioeconomic status and financial disparity of Arab participants may reduce their access to dementia management services, especially in their first language, due to limited availability and transportation barriers in less-developed locations [1,3,4,5,6,7,8]. This explanation may be supported by our finding that Arab patients were diagnosed more frequently by their GP or a neurologist, whereas Jewish patients were diagnosed more commonly by a geriatrician.

Lastly, the higher rates of AD among Arab participants may be partially due to the higher rate of consanguineous marriages among Israeli Arabs, which may increase the genetic predisposition to AD [20,21]. This is potentially supported by the fact that Arab ethnicity remained significantly associated with an AD diagnosis even after adjusting for demographic, educational, socioeconomic, and other clinical risk factors.

We compared our findings on background diagnoses with those of a recent study that examined disparities in chronic disease among CHS-insured patients, similar to our cohort. However, that study did not specify how ethnicity was determined [9]. In both studies, Arab participants had higher rates of diabetes mellitus and obesity, while Jewish participants had higher rates of hypertension, PVD, and depression. In contrast to their findings, we observed higher rates of hyperlipidemia in the Arab group and comparable rates of ischemic heart disease and BMI between the two groups. The average age of participants in that study was approximately thirty years younger than in ours, and it did not specifically focus on dementia patients, which could account for the subtle differences between the two studies.

Depression, a known risk factor for AD [3], was found to be more prevalent among the Jewish population in our study. This aligns with one previous study [9]; however, a different one reported that the depression rate was 2.5 times higher in Arab participants in Israel compared to Jewish participants [22]. That study focused exclusively on the population of Hadera, a mostly urban community, and used the Harvard Department of Psychiatry National Depression Screening Day Scale (HANDS) questionnaire. As this tool has not been validated for this specific population, the results may be influenced by cultural differences. It may be that Jewish patients are more prone to depression because of an urban lifestyle and weaker family as well as community support networks [9,23]. The discrepancy in the findings could also be explained by the underdiagnosis of depression in Arab patients due to stigma, lower awareness, and poorer access to healthcare services in the Arab population [23,24].

Malignancy was more prevalent among the Jewish population, a finding consistent with previous studies as well as the Israel National Cancer Registry [3]. This may result from a more urban lifestyle among the Jewish population, along with potential genetic factors. However, it is also possible that the Arab population is underdiagnosed for malignancy due to their limited access to healthcare, similar to other minorities [1,3,4,5,6,7,8].

Most of the patients included in the study were diagnosed by their GP, with smaller proportions diagnosed by geriatricians or neurologists. A negligible percentage of patients were diagnosed by a pediatrician, likely because many board-certified pediatricians in Israel also serve as GPs for patients of all ages. Notably, Arab participants were more frequently diagnosed by GPs and neurologists, while Jewish participants were more frequently diagnosed by geriatricians. This difference may exist partly because geriatricians in Israel treat the older population (age 65 and older), whereas the average age of Arab patients was five years younger than that of the Jewish patients. Additionally, geriatricians primarily work in specialized clinics concentrated mostly in larger cities in Israel. Therefore, this difference in specialist attendance may reflect poorer access to centralized geriatric clinics, as Arab communities are often located in peripheral areas, leading to greater reliance on other providers. These patterns emphasize that service availability significantly influences diagnostic pathways.

Since identifying reversible causes of dementia is crucial [25], we examined the diagnostic work-ups performed for cognitive decline. Jewish participants were more likely to be tested for thyroid function and vitamin B12 levels. This difference may be partially attributed to differences in diagnostic approaches and healthcare access; while Jewish patients were more often managed by geriatricians, Arab patients relied more on neurologists and GPs. Both groups underwent comparable rates of LDL testing and head imaging.

Another finding was that Arab patients were less inclined to purchase anti-dementia medications in comparison to Jewish patients, despite attending more follow-up visits with neurologists and psychiatrists. Several factors may explain this apparent discrepancy in treatment adherence. Firstly, the lower proportion of MCI diagnoses among Arab participants may indicate a later-stage dementia diagnosis, at which point anti-dementia medications may be less effective or clinically irrelevant. Therefore, lower medication purchase rates in this group may reflect appropriate clinical decision-making rather than non-compliance. Secondly, cultural factors may play a significant role. Caregivers in Arabic households may be reluctant to medicate older adults due to concerns about side effects or fatalism. Additionally, cultural perceptions of dementia as a normal part of aging, compounded by stigma and limited awareness of dementia services, may reduce both demand for and acceptance of treatment [5]. Thirdly, disparities in access to healthcare services of geriatricians, particularly in Arabic, may limit diagnosis and treatment opportunities. Lastly, socioeconomic constraints (as reflected by SES data) and bureaucratic barriers may limit access to subsidized medications or deter patients from navigating complex health systems, especially ones that are not in their first language [26].

Despite the well-documented ethnic disparities in dementia diagnosis and care observed in other societies, research on this topic has been limited in Israel. To the best of our knowledge, our study is the first to directly compare the characteristics of dementia diagnosis and treatment among the two main ethnic groups in Israel in a community setting. Its strengths include a large sample size and comprehensive data derived from recorded medical and administrative records, mitigating recall and selection biases.

However, relying on such data has limitations related to potential inaccuracies. Treatment status was determined by prescription purchases, and to improve accuracy we included only patients who filled at least three prescriptions. The diagnosis was based on ICD-9 coding without reviewing individual medical records. Thus, the study explored dementia diagnosis rather than the actual incidence of dementia.

Several factors can influence diagnostic accuracy. Overdiagnosis may be due to insurance issues, legal demands for medical care, or variability in physician screening and diagnostic approaches. Underdiagnosis may occur due to cultural beliefs that cognitive deterioration is part of normal aging and disbelief in dementia treatment options [3]. Our study indicates that a higher percentage of Arab participants were diagnosed by GPs and neurologists compared to Jewish participants, which may influence the approach to dementia diagnosis.

Another limitation of our study is the use of residential address as a proxy for ethnicity. The use of geocoding as a proxy for ethnicity and socioeconomic status in places of ethnic segregation is a widely accepted methodology in epidemiological research, particularly in large database studies where self-reported data are absent [27,28]. To reduce this risk of ethnic misclassification, we analyzed only populations that, according to CBS, reside in localities where at least 99% of the population is identified as either exclusively Arab or exclusively Jewish. We deliberately excluded cities with mixed or ambiguous ethnic compositions. While this step reduced the overall sample size, it significantly enhanced the accuracy of our ethnic categorization. We recognize, however, that any proxy method carries an inherent risk of error. Therefore, future research should aim to validate these classifications by using direct, patient-reported ethnicity.

The lack of clinical data, such as MMSE scores or degree of functional impairment, is another limitation of our study. This study was designed to use administrative ICD-9 codes rather than individual chart reviews. It enabled us to include a much larger and more representative sample, enhancing the statistical power and generalizability of our findings. While this design prevents the assessment of disease severity or diagnostic delay, the observed ethnic differences in diagnostic patterns, treatment, and follow-up remain robust and informative at the population level.

Finally, this study is a descriptive study in nature; hence, residual confounding remains of concern.

In summary, our study identified significant ethnic disparities in dementia prevalence, diagnosis, work-up, and treatment in Israel. These disparities are likely rooted in a complex interplay of lifestyle, education, socioeconomic status, and healthcare access, and they carry profound implications for healthcare policy, clinical practice, and the ethical imperative to foster health equity.

Addressing these factors is crucial on both the personal and establishment levels. On a policy level, our findings call for a strategic reassessment of resource allocation to dismantle the systemic barriers affecting underserved populations, particularly those in peripheral regions. This requires developing and funding culturally tailored public health campaigns, improving healthcare infrastructure in remote areas, and ensuring the consistent availability of services in a patient’s first language. In the clinical setting, healthcare providers require structured training to recognize how diverse cultural backgrounds influence symptom presentation and treatment compliance. Furthermore, encouraging interdisciplinary collaboration among neurologists, geriatricians, and primary care physicians is crucial for improving diagnostic accuracy and continuity of care. Implementing these steps is essential to ensure equitable, respectful care, and should guide future healthcare planning and professional training.

In conclusion, our study showed that the Arab population is diagnosed with AD and VD more frequently and at a younger age than their Jewish counterparts. Understanding the complex sociocultural and systemic factors driving these disparities is crucial for creating targeted interventions, advancing early detection, and ultimately achieving greater healthcare equity.

## Figures and Tables

**Table 1:** Demographic data.

	Jewish Participants (*n* = 8467)	Arab Participants (*n* = 6275)	*p* Value
Gender (male)	3646 (43.1%)	2690 (42.9%)	0.815
Age at diagnosis (SD)	80.150 (9.454)	75.783 (10.276)	<0.001
BMI and Lifestyle			
Performing physical activity *	1334 (20.9%)	550 (10.2%)	<0.001
Positive smoking history (current/past) **	2027 (25.8%)	1821 (30%)	<0.001
BMI (SD) ***	27.094 (5.227)	28.477 (6.090)	<0.001
Background Diagnoses			
Hypertension	4714 (55.7%)	3218 (51.3%)	<0.001
Diabetes	3009 (35.5%)	2970 (47.3%)	<0.001
Ischemic heart disease (IHD)	1896 (22.4%)	1385 (22.1%)	0.643
Peripheral vascular disease (PVD)	348 (4.1%)	184 (2.9%)	<0.001
Stroke (CVA)	2815 (33.2%)	2142 (34.1%)	0.259
Intracerebral hemorrhage	1977 (23.3%)	1879 (29.9%)	<0.001
History of malignancy	2356 (27.8%)	687 (10.9%)	<0.001
Hyperlipidemia	2881 (34.0%)	2390 (38.1%)	<0.001
Extrapyramidal disorders	1233 (14.6%)	472 (7.5%)	<0.001
Essential tremor	128 (1.5%)	68 (1.1%)	0.025
Repeated falls	1501 (17.7%)	639 (10.2%)	<0.001
Vision problems	2965 (35.0%)	2337 (37.2%)	0.005
Hearing problems	1545 (18.2%)	705 (11.2%)	<0.001
Sleep apnea	120 (1.4%)	67 (1.1%)	0.061
Depression	2417 (28.5%)	985 (15.7%)	<0.001
Anxiety	307 (3.6%)	114 (1.8%)	<0.001
Psychosis	476 (5.6%)	242 (3.9%)	<0.001

*: 2094 Jewish participants and 869 Arab participants had missing data. **: 587 Jewish participants and 208 Arab participants had missing data. ***: 1453 Jewish participants and 881 Arab participants had missing data. GP: general practitioner.

**Table 2:** Diagnosis and work-up.

	Jewish Participants (*n* = 8467)	Arab Participants (*n* = 6275)	*p*-Value
Diagnosis			
AD *	2524 (29.8%)	2387 (38.0%)	<0.001
MCI *	2227 (26.3%)	647 (10.3%)	<0.001
VD *	540 (6.4%)	789 (12.6%)	<0.001
FTD *	17 (0.2%)	9 (0.1%)	0.412
PD *	36 (0.4%)	20 (0.3%)	0.299
LBD *	7 (0.1%)	3 (0.0%)	0.421
CJD *	14 (0.2%)	3 (0%)	17 (0.1%)
Huntington’s disease	7 (0.1%)	8 (0.1%)	15 (0.1%)
Dementia NOS	3086 (36.4%)	2400 (38.2%)	5486 (37.2%)
Diagnosed By			
GP	3385 (68.2%)	2630 (76.1%)	<0.001
Neurologist	388 (7.8%)	397 (11.5%)	<0.001
Psychiatrist	18 (0.4%)	13 (0.4%)	<0.001
Geriatrician	876 (17.7%)	249 (7.2%)	<0.001
Pediatrician	215 (4.3%)	102 (3.0%)	<0.001
Work-Up			
TSH	8045 (95.0%)	5354 (85.3%)	<0.001
T4	5133 (60.6%)	3293 (52.5%)	<0.001
Vitamin B12	7158 (84.5%)	3938 (62.8%)	<0.001
LDL	7920 (93.5%)	5855 (93.3%)	0.572
HBA1C	5900 (69.7%)	4737 (75.5%)	<0.001
CT/MRI	4282 (50.6%)	3092 (49.3%)	0.119

* AD: Alzheimer’s disease; FTD: frontotemporal dementia; PD: Parkinson’s disease; LBD: Lewy body dementia; MCI: mild cognitive impairment; VD: vascular dementia; and CJD: Creutzfeldt–Jakob disease.

**Table 3:** Follow-up and dementia treatment.

	Jewish Participants (*n* = 8467)	Arab Participants (*n* = 6275)	*p*-Value
Visits to neurologist per year (SD)	1.502 (2.702)	1.973 (3.084)	<0.001
Visits to psychiatrist per year (SD)	2.002 (3.179)	2.707 (3.825)	<0.001
Visits to geriatrician per year (SD)	1.037 (2.164)	0.893 (2.088)	<0.077
Overall follow-up (years)	6.805 (5.240)	5.714 (4.909)	<0.001
Three and more purchases of anti-dementia treatment	2231 (82.4%)	893 (73.0%)	<0.001
Three and more purchases of antipsychotic medication	1634 (63.5%)	1350 (62.1%)	0.360

**Table 4:** Socioeconomic data.

	Jewish Participants (*n* = 8467)	Arab Participants (*n* = 6275)	*p*-Value
Education years (SD)	13.917 (1.056)	12.044 (0.633)	<0.001
Percentage of academic degree holders (SD)	43.284 (22.338)	19.990 (6.162)	<0.001
Socioeconomic status (SES) (SD)	6.436 (3.126)	3.237 (1.254)	<0.001
Average monthly income in shekels (SD)	8082.212 (3788.062)	4094.808 (947.784)	<0.001

## Data Availability

Due to patient privacy and ethical considerations, the data are not publicly available. The data supporting the findings of this study are available upon request from the Clalit Research Department, provided that Clalit’s security standards are met.

## Supplementary Materials

The following supporting information can be downloaded at: https://www.mdpi.com/article/10.3390/jcm14175926/s1, Table S1: Multivariable logistic regression, after adjusting for demographic data for Alzheimer disease (AD) diagnosis, *n* = 14,742; Table S2: Multivariable logistic regression, after adjusting for demographic data and residential as well as socioeconomic characteristics of Alzheimer disease (AD) diagnosis; *n* = 6393; Table S3: Multivariable logistic regression, after adjusting for demographic data for vascular dementia (VD) diagnosis, *n* = 14,742; and Table S4: Multivariable logistic regression, after adjusting for demographic data and residential as well as socioeconomic characteristics of vascular dementia (VD) diagnosis; *n* = 6393.
