# Supplementary material for: Ethnic Differences in Dementia Diagnosis and Treatment in Israel
Source: J Clin Med. 2025 Aug 22;14(17):5926. doi: 10.3390/jcm14175926 (PMC12429298; doi:10.3390/jcm14175926)
Supplement: Supplementary file 1 [file jcm-14-05926-s001.zip › jcm-3732887-supplementary.pdf]

Supplemental material

Table S1: Multivariable logistic regression, after adjusting for demographic data for Alzheimer disease (AD) diagnosis, N=14,742

**Characteristic**	**OR**[1]	**95% CI**[1]	**p-value**
Ethnicity			
Jewish	—	—	
Arab	**1.75**	1.62, 1.89	<0.001
Gender			
Female	—	—	
Male	0.93	0.86, 1.01	0.082
Reference Event-Age at diagnosis	1.05	1.04, 1.05	<0.001
Smoking status			
Non smoker	—	—	
Past smoker	0.92	0.83, 1.02	0.12
Current smoker	0.90	0.79, 1.02	0.11
Hypertension	0.99	0.92, 1.08	0.9
Diabetes	1.03	0.95, 1.11	0.5
Ischemic Heart Disease (IHD)	0.99	0.90, 1.09	0.9

Characteristic	OR[1]	95% CI[1]	p-value
Peripheral Vascular Disease (PVD)	1.00	0.83, 1.22	>0.9
Stroke (CVA)	0.93	0.85, 1.02	0.11
Intracerebral hemmorhage	0.95	0.86, 1.04	0.3
Malignancy	0.95	0.87, 1.04	0.3
Hyperlipidemia	1.05	0.96, 1.14	0.3
Extrapyramidal Disorders	1.31	1.17, 1.46	<0.001
Essential tremor	0.73	0.52, 1.00	0.058
Repeated falls	0.80	0.72, 0.89	<0.001
Vision Problems	0.88	0.81, 0.94	<0.001
Hearing Problems	0.81	0.73, 0.90	<0.001
Sleep apnea	0.71	0.50, 1.01	0.063
Depression	0.97	0.89, 1.06	0.5
Anxiety	0.95	0.76, 1.17	0.6
Psychosis	1.08	0.92, 1.27	0.4

Table S2: Multivariable logistic regression, after adjusting for demographic data and residential and socioeconomic characteristics for Alzheimer disease (AD) diagnosis; N=6,393

Characteristic	OR[1]	95% CI[1]	p-value
Ethnicity			
Jewish	—	—	
Arab	1.73	1.40, 2.13	<0.001
Gender			
Female	—	—	
Male	0.88	0.77, 0.99	0.040
Reference Event-Age at diagnosis	1.05	1.04, 1.05	<0.001
BMI	1.00	0.99, 1.01	0.8
Smoking status			
Non smoker	—	—	
Past smoker	1.06	0.90, 1.25	0.5

**Characteristic**	**OR**[1]	**95% CI**[1]	**p-value**
Current smoker	0.93	0.78, 1.10	0.4
SES	1.16	1.05, 1.28	0.004
AVERAGE YEARS OF SCHOOLING, OF AGED 25-54	1.01	0.88, 1.16	0.9
AVERAGE MONTHLY INCOME PER CAPITA	1.00	1.00, 1.00	0.008
Hypertension	0.88	0.78, 0.99	0.040
Diabetes	1.09	0.97, 1.22	0.2
Ischemic Heart Disease (IHD)	1.00	0.86, 1.15	>0.9
Peripheral Vascular Disease (PVD)	1.21	0.87, 1.66	0.3
Stroke (CVA)	1.02	0.89, 1.17	0.8
Intracerebral hemmorhage	0.97	0.84, 1.11	0.6
Malignancy	0.97	0.82, 1.14	0.7

**Characteristic**	**OR**[1]	**95% CI**[1]	**p-value**
Hyperlipidemia	1.02	0.90, 1.16	0.8
Extrapyramidal Disorders	1.26	1.04, 1.52	0.016
Essential tremor	1.03	0.63, 1.65	>0.9
Repeated falls	0.82	0.69, 0.96	0.018
Vision Problems	0.95	0.85, 1.06	0.4
Hearing Problems	0.95	0.80, 1.11	0.5
Sleep apnea	0.81	0.48, 1.31	0.4
Depression	0.91	0.79, 1.04	0.2
Anxiety	0.94	0.65, 1.34	0.7
Psychosis	1.27	0.98, 1.64	0.067

Table S3: Multivariable logistic regression, after adjusting for demographic data for Vascular Dementia VD diagnosis, N=14,742

Characteristic	OR[1]	95% CI[1]	p-value
Ethnicity			
Jewish	—	—	
Arab	1.35	1.18, 1.53	<0.001
Gender			
Female	—	—	
Male	0.92	0.80, 1.05	0.2
Reference Event-Age at diagnosis	0.91	0.91, 0.92	<0.001
Smoking status			
Non smoker	—	—	
Past smoker	0.79	0.65, 0.95	0.015
Current smoker	1.13	0.94, 1.35	0.2
Hypertension	0.95	0.82, 1.09	0.4
Diabetes	0.82	0.71, 0.94	0.004
Ischemic Heart Disease (IHD)	1.18	1.00, 1.39	0.049

Characteristic	OR[1]	95% CI[1]	p-value
Peripheral Vascular Disease (PVD)	1.42	1.03, 1.92	0.028
Stroke (CVA)	1.08	0.92, 1.27	0.3
Intracerebral hemmorhage	0.89	0.75, 1.05	0.2
malignancy	0.83	0.69, 0.99	0.038
Hyperlipidemia	0.92	0.80, 1.07	0.3
Extraphyramidal Disorders	0.63	0.49, 0.79	<0.001
Essential tremor	1.04	0.55, 1.80	0.9
Repeated falls	0.86	0.70, 1.06	0.2
Vision Problems	1.40	1.23, 1.59	<0.001
Hearing Problems	0.88	0.73, 1.06	0.2
Sleep apnea	0.78	0.42, 1.33	0.4
Depression	0.85	0.73, 0.99	0.037
Anxiety	0.70	0.44, 1.06	0.11
Psychosis	0.66	0.47, 0.91	0.014

Table S4: Multivariable logistic regression, after adjusting for demographic data and residential and socioeconomic characteristics for Vascular Dementia (VD) diagnosis; N=6,393

**Characteristic**	**OR**[1]	**95% CI**[1]	**p-value**
Ethnicity			
Jewish	—	—	
Arab	1.39	0.96, 2.03	0.087
Gender			
Female	—	—	
Male	1.14	0.92, 1.40	0.2
Reference Event-Age at diagnosis	0.89	0.88, 0.90	<0.001
BMI	1.02	1.00, 1.03	0.027
Smoking status			
Non smoker	—	—	
Past smoker	0.69	0.51, 0.92	0.013

Characteristic	OR[1]	95% CI[1]	p-value
Current smoker	1.07	0.83, 1.39	0.6
SES	0.96	0.80, 1.15	0.7
AVERAGE YEARS OF SCHOOLING, OF AGED 25-54	0.92	0.74, 1.16	0.5
AVERAGE MONTHLY INCOME PER CAPITA	1.00	1.00, 1.00	0.6
Hypertension	0.89	0.73, 1.09	0.3
Diabetes	0.75	0.62, 0.91	0.004
Ischemic Heart Disease (IHD)	1.06	0.82, 1.36	0.7
Peripheral Vascular Disease (PVD)	1.09	0.64, 1.81	0.7
Stroke (CVA)	1.10	0.86, 1.41	0.4
Intracerebral hemmorhage	0.67	0.52, 0.87	0.003
Malignancy	0.84	0.61, 1.14	0.3

Characteristic	OR[1]	95% CI[1]	p-value
Hyperlipidemia	0.93	0.75, 1.15	0.5
Extrapyramidal Disorders	0.36	0.22, 0.55	<0.001
Essential tremor	1.36	0.54, 3.00	0.5
Repeated falls	0.46	0.30, 0.67	<0.001
Vision Problems	1.53	1.28, 1.83	<0.001
Hearing Problems	0.82	0.62, 1.07	0.2
Sleep apnea	0.80	0.33, 1.70	0.6
Depression	0.89	0.70, 1.11	0.3
Anxiety	0.50	0.22, 0.99	0.068
Psychosis	0.46	0.26, 0.76	0.004
